# Long-term quality of life after surgery of head and neck cancer with microvascular reconstruction: a prospective study with 4.9-years follow-up

**DOI:** 10.1007/s10006-019-00806-w

**Published:** 2019-11-05

**Authors:** Satu Kainulainen, A. M. Koivusalo, R. P. Roine, T. Wilkman, H. Sintonen, J. Törnwall, H. Thorén, P. Lassus

**Affiliations:** 1 Department of Oral and Maxillofacial Surgery, Helsinki University Hospital, University of Helsinki, Helsinki, Finland; 2 Department of Oral and Maxillofacial Surgery, Helsinki University Hospital, P.O. Box 220, FI-00029 HUS, Helsinki, Finland; 3 Department of Anesthesia and Intensive Care Unit, Helsinki University Hospital, University of Helsinki, Helsinki, Finland; 4 Department of Health and Social Management, University of Eastern Finland, Kuopio, Finland; 5 Group Administration, Helsinki University Hospital, University of Helsinki, Helsinki, Finland; 6 Department of Public Health, University of Helsinki, Helsinki, Finland; 7 University of Helsinki, Helsinki, Finland; 8 Department of Oral and Maxillofacial Diseases, Institute of Dentistry, University of Turku and Turku University Hospital, Turku, Finland; 9 Department of Plastic Surgery, Helsinki University Hospital, Helsinki, Finland

**Keywords:** Head and neck cancer, Health-related quality of life, Microvascular reconstruction, Surgery

## Abstract

**Purpose:**

The aim of this study was to evaluate the long-term health-related quality of life (HRQoL) of head and neck cancer patients with microvascular surgery. Surgical treatment causes great changes in patient HRQoL. Studies focusing on long-term HRQoL after microvascular reconstruction for head and neck cancer patients are scarce.

**Methods:**

We conducted a prospective study of 93 patients with head and neck cancer and microvascular reconstruction in Helsinki University Hospital Finland. HRQoL was measured using the 15D instrument at baseline and after a mean 4.9-years follow up. Results were compared with those of an age-standardized general population.

**Results:**

Of the 93 patients, 61 (66%) were alive after follow-up; of these, 42 (69%) answered the follow-up questionnaire. The median time between surgery and HRQoL assessment was 4.9 years (range 3.7–7.8 years). The mean 15D score of all patients (*n* = 42) at the 4.9-years follow up was statistically significantly (*p* = 0.010) and clinically importantly lower than at baseline. The dimensions of “speech” and “usual activities” were significantly impaired at the end of follow up. There was a significant difference at the 4.9-years follow-up in the mean 15D score between patients and the general population (*p* = 0.014). After follow up, patients were significantly (*p* < 0.05) worse off on the dimensions of “speech,” “eating,” and “usual activities.”

**Conclusions:**

Long-term HRQoL was significantly reduced in the whole patient cohort. Speech and usual activities were the most affected dimensions in head and neck cancer patients with microvascular reconstruction at the end of the 4.9-years follow up.

## Introduction

The incidence of head and neck cancer is increasing in Finland. In 2015 the incidence of oropharyngeal cancer in Finland reached approximately 700 cases out of a population of 5.5 million. Most cases were in men [1]. Malignant tumors in the head and neck area often require microvascular reconstruction to restore the surgical defect. Surgery for head and neck cancer and possible oncological treatments are associated with significant physiological and psychological disruption of life due to physical, esthetic, and functional disability [2].

Health-related quality of life (HRQoL) has become an important instrument to measure the outcome of head and neck cancer patients. There are many disease-specific and generic questionnaires to measure HRQoL in these populations [3–6]. HRQoL after surgical or oncological treatment of head and neck cancers is well studied. According to previous studies, advanced tumors, extensive surgical resection, free-flap reconstruction, and postoperative radiotherapy are associated with low HRQoL [7–9]. Studies focusing on HRQoL after microvascular reconstruction for head and neck cancers are scarce. The limitations of these previous studies include an often short-term follow-up period and variability in HRQoL instruments.

The aim of this prospective cohort study was to evaluate the long-term HRQoL of head and neck cancer patients with microvascular reconstruction compared to an age- and gender-standardized sample of the general population. HRQoL was measured using the generic 15-dimensional (15D) instrument, which is a multidimensional generic HRQoL instrument.

## Materials and methods

Between December 2008 and February 2013, we conducted a randomized double-blind controlled trial for patients with head and neck cancer requiring microvascular reconstruction at the Department of Oral and Maxillofacial Surgery and the Department of Plastic Surgery, Helsinki University Hospital. Patients with head and neck cancer who had a microvascular reconstruction were included in the study and evaluated by the multidisciplinary head and neck tumor board of the Helsinki University Hospital. The original purpose of the study was to evaluate the effects of dexamethasone on recovery after microvascular surgery. A total of 93 patients were included in the study, 73 from the Department of Maxillofacial Surgery and 20 from the Department of Plastic Surgery. Fifty-one patients received dexamethasone (DEX) and 42 patients did not (NON-DEX). A total dose of 60 mg dexamethasone was administered to 51 patients over 3 days peri- and postoperatively. A detailed description of the study protocol and patient characteristics is found in our previous study [10]. The study was approved by the Research Ethical Board of Helsinki University Central Hospital, Finland. Informed consent was obtained from all individual participants included in the study before randomization. HRQoL was obtained from all patients, despite randomization.

In this study, HRQoL of the patients was measured with the multidimensional, generic 15D instrument. Although there is no consensus for the preferred instrument, the 15D has been used in many cancer patient groups [11], including those with head and neck cancer [12, 13]. The 15D compares favorably with other generic HRQoL instruments such as the NHP, SF-20, SF-6D, and EQ-5D. A recent study ranked the 15D first among the most frequently used generic HRQoL instruments in sensitivity and construct validity in the disease area of cancer [14]. Using the 15D also enables comparison of HRQoL results with an age-standardized general population. The 15D questionnaire is designed for populations aged over 15 years.

The 15 dimensions of the instruments are: moving, seeing, hearing, breathing, sleeping, eating, speech, excretion, usual activities, mental function, discomfort and symptoms, depression, distress, vitality, and sexual activity. For each dimension, the respondent chooses one of the five levels that best describe his/her state of health at the moment (the best level = 1; the worst level = 5). The 15D can be used as both a profile measure and a single index score measure. The single index number (15D score) ranges from 0 (being dead) to 1 (full health). The 15D score is calculated from the health state descriptive system [15]. A change or difference of ± 0.015 in the 15D score is considered clinically important [16–19].

The 15D data for the general population came from the representative National Health 2011 Survey [20]. For comparison with patients, individuals were selected from the Helsinki University Hospital catchment area who were in the age range of the patients (*n* = 1148). This sample was weighted to reflect the age and gender distribution of the patients.

All patients completed the baseline 15D questionnaire before surgery. Follow-up questionnaires were sent to all patients alive in a prepaid, pre-addressed envelope in October 2016. All patients who responded were included in the analysis. The influence of tumor site, use of free flap, tumor stage, and postoperative radiation therapy on long-term HRQoL were investigated. Patient and tumor characteristics are listed in Table 1.

**Table 1:** Patient and tumor characteristics

**Variable**	All patients (n = 42)
Age (years)	66 (39–88)
Follow-up time (years)	4.9 (3.7–7.8)
Gender	
Female/Male	14/28
Smoking (yes/no)	15/27
Reconstruction type	
ALT	16
RFA	26
Reconstruction site	
Maxilla	8
Mandible	6
Tongue	13
Floor of the mouth	7
Tonsilla	2
Cheek	5
Larynx	1
Tumor type	
Epidermoid carcinoma	37
Other	5
T score	
T1–2	26
T3-4A	16
Postoperative radiation therapy (yes/no)	17/25

ALT: Anterolateral thigh perforator flap

RFA: Radial forearm flap

Data given as median and range

## Statistical analysis

Statistical analysis was performed using SPSS for Windows statistical software version 22 (SPSS, Inc., Chicago, IL, USA). The statistical significance of the change in the mean dimension and HRQoL scores was tested by paired samples *t* test. The groups were compared using Chi-square test, independent samples *t* test, or ANOVA where appropriate. Two-sided *p*-values <0.05 were considered statistically significant.

## Results

**Fig. 1:**
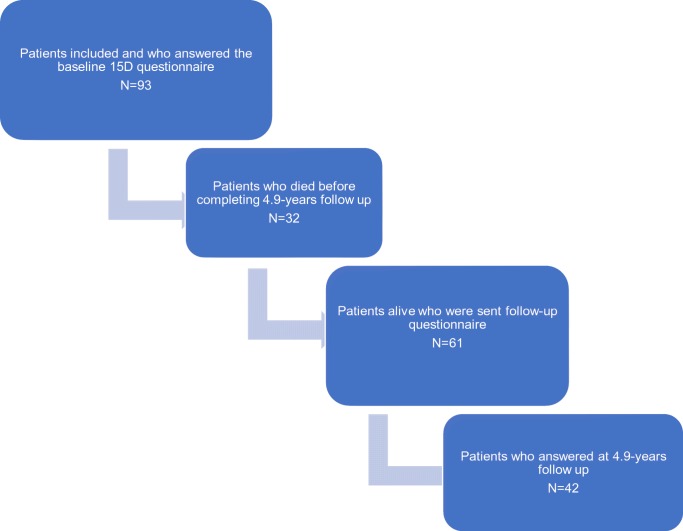
Patient selection process

**Fig. 2:**
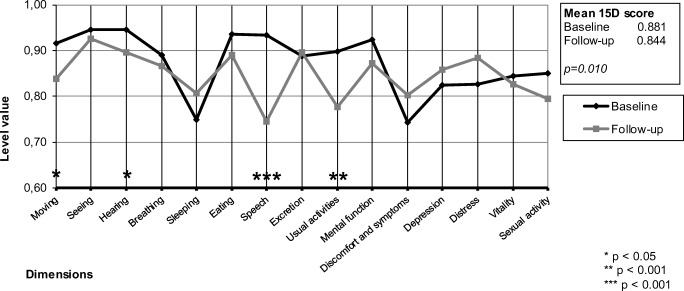
Mean 15D profiles of head and neck cancer patients with microvascular reconstruction (*n* = 42) at baseline and 4.9-years after operation

The median time between surgery and HRQoL assessment was 4.9 years (range 3.7–7.8 years). Of the 93 patients, 61 (66%) were alive by the end of the follow up (December 2016). A total of 42 (69%) patients answered the long-term follow-up questionnaire (Fig. 1). All patients were considered as a one group, independent of dexamethasone administration. The mean 15D score of all patients (*n* = 42) at the 4.9-years follow-up point (0.844) was statistically significantly (*p* = 0.010) and clinically importantly lower than at baseline (0.881). As shown in Fig. 2, patients perceptions of “speech” (*p* < 0.001), “usual activities” (*p* < 0.01), “moving” (*p* < 0.05) and “hearing” (*p* < 0.05) were significantly poorer at the end of follow-up. However, there was some improvement on the psychosocial dimensions of “discomfort and symptoms,” “depression,” and “distress,” although the latter differences were not statistically significant.

**Fig. 3:**
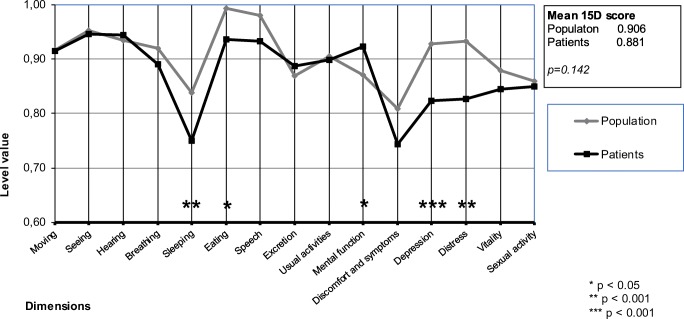
15D profiles of microvascular reconstruction patients at baseline compared with age- and gender-matched general population

**Fig. 4:**
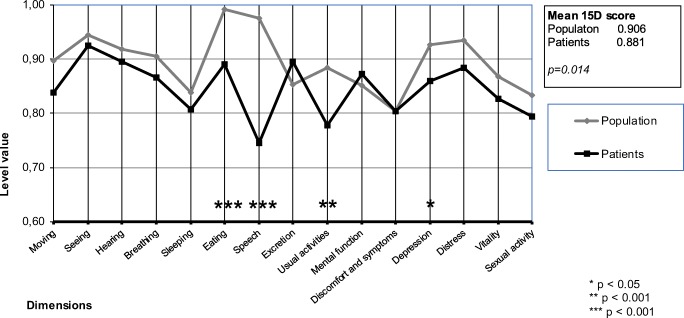
15D profiles of microvascular reconstruction patients at 4.9-years follow up compared with age- and gender-matched general population

Figure 3 shows the preoperative and Fig. 4 the 4.9-years postoperative mean dimension scores relative to the general population. The mean total 15D scores did not differ statistically significantly at baseline (patients 0.881 vs population 0.906, *p* = 0.142). However, dimension scores for “depression,” “mental,” and “distress” were lower in the patient cohort group (*p* < 0.05). There was a significant difference at the 4.9-years follow up in the mean 15D score between patients and the general population (patients 0.844 vs population 0.894, *p* = 0.014). After the 4.9-years follow up, patients were significantly (*p* < 0.05) worse off in the dimensions of “speech,” “eating,” and “usual activities.”

The mean 15D score deteriorated in all patients in the same way regardless of whether or not postoperative radiotherapy was administered. The type of reconstruction (ALT, *n* = 16, 0.807 vs RFA, *n* = 26, 0.867; *p* = 0.122), tumor site (maxilla, 0.837, mandible, 0.915, tongue, 0.865, floor of the mouth, 0.781, tonsillar, 0.773, cheek, 0.866, larynx, 0.671; *p* = 0.378) and tumor score (T1 + 2, 0.863, T3 + 4A, 0.813; *p* = 0.241) had no statistically significant effect on the follow-up 4.9-years mean 15D score (Table 1). Patients with stage T1–2 tumor (n = 26) were significantly better off on the dimensions of “usual activities” (*p* < 0.05), “discomfort and symptoms” (*p* < 0.01), and “sexual activity” (*p* < 0.05) as compared to those with stage a T3 or T4A tumor (*n* = 16) at baseline. The dimension scores of “usual activities” and “hearing” were significantly higher in the T1–2 group than in the T3-4A group at follow up (both *p* < 0.05). Smokers had a statistically significantly lower 15D score both at baseline (0.911 vs. 0.827, *p* = 0.010) and at follow-up (0.890 vs. 0.761; *p* < 0.001) than non-smokers; in particular the dimensions of “speech” and “usual activities” were impaired at follow-up.

## Discussion

In this prospective study, HRQoL was assessed using the 15D instrument after a 4.9-years follow-up in 42 patients with head and neck cancer and microvascular reconstruction. The response rate was 69%, which can be considered good. The primary aim of this study was to evaluate the long-term HRQoL of head and neck cancer patients with microvascular reconstruction. The advantage of the study is the prospective study design in a coherent group of head and neck cancer patients with a single treatment modality.

The 15D is a widely used HRQoL instrument that enables comparison between head and neck cancer patients with free flap and the general population. Several studies have investigated HRQoL after treatment of oropharyngeal cancer, particularly oncological treatment, and showed that the 15D is a useful instrument for evaluation in these patients [12, 13]. Those studies show that, as expected, a decline in HRQoL is usually seen during the first 3 months but then HRQoL gradually improves towards 1 year [21]. Chen et al. noted that even after almost 5 years after oncological treatment, patients are satisfied with their quality of life (QoL) [22].

There are surprisingly few studies published on long-term HRQoL after microvascular reconstruction surgery of head and neck cancer patients. Pierre et al. showed in their prospective study of 64 patients that long-term QoL after oncologic surgery and microvascular free-flap reconstruction in patients with oral cancer is satisfactory [8]. Bozec et al. studied long-term QoL and psychosocial outcomes after oropharyngeal cancer surgery and radial forearm free-flap reconstruction and observed that long-term QoL was well-preserved [23]. In our study, the mean 15D score at the 4.9-years follow-up point was significantly and clinically importantly lower than at baseline.

Speech problems are common after surgery of head and neck cancer. The dimensions of “speech” and “usual activities” were the most affected dimensions at the 4.9-years follow-up (*p* < 0.001). Psychological distress (swallowing and speech problems, changed appearance, fear of recurrence and death) is common, even long after treatment in head and neck cancer patients [24]. Although the differences were not statistically significant, in the present study the dimension of “discomfort and symptoms” and the psychological dimensions of “depression” and “distress” interestingly improved during long-term follow up. If any anxiety occurs during the treatment period or follow-up visits, patients will have access to psychotherapy in our hospital. This may improve depression and the continuity of treatment. Regular assessments are beneficial for mental health.

Large tumors of the head and neck area are associated with a poor prognosis. Patients with advanced tumor extension (tumor stage) disease often need more complex resection and surgery, which affects postoperative HRQoL more than the treatment of those with early-stage disease [25]. Some studies have shown that advanced tumor stage, oropharyngeal cancer, and surgery in combination with chemoradiotherapy impact overall HRQoL negatively compared to early stage, oral cavity cancer, and surgery only [26, 27]. In our study, the dimension scores of “usual activities” and “hearing” were significantly higher in the early-stage T1–2 group than in the T3-4A group at follow-up (both *p* < 0.05). Postoperative radiation therapy causes damage to tissues such as the salivary gland, which can have a major impact on HRQoL [28]. However, postoperative radiation therapy did not impair HRQoL in our patients (*p* = 0.8) compared with patients who were treated with surgery only. Although there was no significant difference in tumor site and long-term HRQoL in our research, the size of the study is probably not enough to differentiate between the different localizations and it would require a separate broader and possibly a multi-center study. Although the number of patients in this study was small, the strength of the study is that it is prospective, thus minimizing the effect of bias. The follow-up evaluation was conducted over an extended period (range 3.7–7.8 years) and the response rate was good (69%).

## Conclusion

We observed in this study that speech and usual activities were the most affected dimensions in head and neck cancer patients with microvascular reconstruction at the end of the 4.9-years follow-up. Long-term HRQoL was significantly reduced in the whole patient cohort.
